# Chloridobis[2-(1,3-thia­zol-4-yl-κ*N*)-1*H*-benzimidazole-κ*N* ^3^]cobalt(II) chloride dihydrate

**DOI:** 10.1107/S1600536812030371

**Published:** 2012-07-10

**Authors:** Zhan-Wang Shi, Yan Qin, Yan-Xia Lin, Wei Wu, Peng Liang

**Affiliations:** a Department of Chemistry and Chemical Engineering, Guangxi University for Nationalities, Nanning 530006, People’s Republic of China; b Hybio Pharmaceutical Co. Ltd, Shenzhen 518057, People’s Republic of China

## Abstract

In the title compound, [CoCl(C_10_H_7_N_3_S)_2_]Cl·2H_2_O, the Co^II^ atom is five-coordinated by four N atoms from two chelating 2-(1,3-thia­zol-4-yl)-1*H*-benzimidazole ligands and one Cl atom in a distorted trigonal–bipyramidal geometry. In the crystal, N—H⋯O and O—H⋯Cl hydrogen bonds and π–π inter­actions between the thia­zole, imidazole and benzene rings [centroid-to-centroid distances 3.546 (2), 3.683 (2) and 3.714 (2) Å] link the complex cations, chloride anions and uncoordinating water mol­ecules into a three-dimensional network.

## Related literature

For related structures, see: Devereux *et al.* (2004 ▶, 2007 ▶); Flores-Alamo *et al.* (2010 ▶); Jean *et al.* (2002 ▶); Mothilal *et al.* (2004 ▶); Murugesan *et al.* (1998 ▶); Ren *et al.* (2010 ▶); Stanley *et al.* (2002 ▶); Trus & Marsh (1973 ▶); Wisniewski *et al.* (2001 ▶).
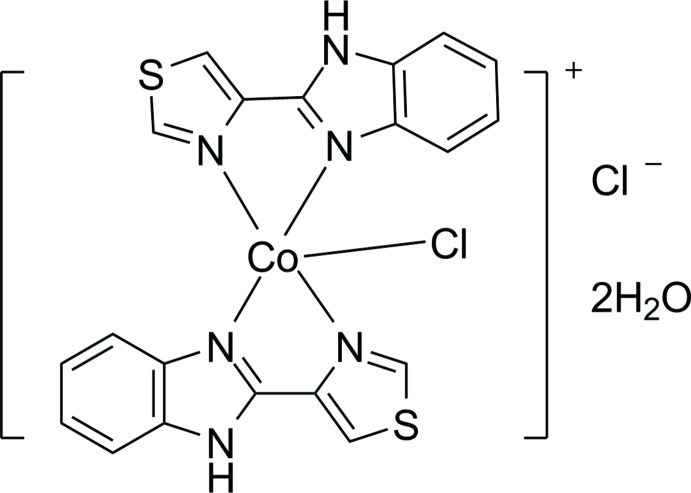


## Experimental

### 

#### Crystal data

[CoCl(C_10_H_7_N_3_S)_2_]Cl·2H_2_O
*M* *_r_* = 568.35
Monoclinic, 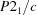

*a* = 14.803 (4) Å
*b* = 11.709 (3) Å
*c* = 14.082 (4) Å
β = 101.439 (4)°
*V* = 2392.3 (11) Å^3^
*Z* = 4
Mo *K*α radiation
μ = 1.15 mm^−1^
*T* = 296 K
0.20 × 0.20 × 0.20 mm

#### Data collection

Bruker SMART 1000 CCD diffractometer
Absorption correction: multi-scan (*SADABS*; Sheldrick, 1996 ▶) *T*_min_ = 0.803, *T*_max_ = 0.803
12683 measured reflections
4200 independent reflections
3517 reflections with *I* > 2σ(*I*)
*R*_int_ = 0.029

#### Refinement

*R*[*F* ^2^ > 2σ(*F* ^2^)] = 0.033
*wR*(*F* ^2^) = 0.096
*S* = 1.05
4200 reflections
298 parameters
H-atom parameters constrained
Δρ_max_ = 0.52 e Å^−3^
Δρ_min_ = −0.37 e Å^−3^

### 

Data collection: *SMART* (Bruker, 2007 ▶); cell refinement: *SAINT* (Bruker, 2007 ▶); data reduction: *SAINT*; program(s) used to solve structure: *SHELXS97* (Sheldrick, 2008 ▶); program(s) used to refine structure: *SHELXL97* (Sheldrick, 2008 ▶); molecular graphics: *DIAMOND* (Brandenburg, 1999 ▶); software used to prepare material for publication: *SHELXTL* (Sheldrick, 2008 ▶).

## Supplementary Material

Crystal structure: contains datablock(s) I, global. DOI: 10.1107/S1600536812030371/hy2563sup1.cif

Structure factors: contains datablock(s) I. DOI: 10.1107/S1600536812030371/hy2563Isup2.hkl

Supplementary material file. DOI: 10.1107/S1600536812030371/hy2563Isup3.cdx

Additional supplementary materials:  crystallographic information; 3D view; checkCIF report

## Figures and Tables

**Table 1:** Hydrogen-bond geometry (Å, °)

*D*—H⋯*A*	*D*—H	H⋯*A*	*D*⋯*A*	*D*—H⋯*A*
N3—H3⋯O2^i^	0.86	1.90	2.748 (3)	169
N6—H6⋯O1	0.86	1.86	2.704 (3)	168
O1—H1*B*⋯Cl2^ii^	0.85	2.27	3.104 (3)	169
O1—H1*A*⋯Cl1^iii^	0.85	2.43	3.272 (3)	169
O2—H2*B*⋯Cl1	0.85	2.45	3.296 (3)	171
O2—H2*A*⋯Cl2	0.85	2.21	3.052 (2)	174

Symmetry codes: (i) 
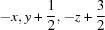
; (ii) 
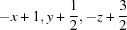
; (iii) 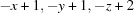.

**Fig. 1.**
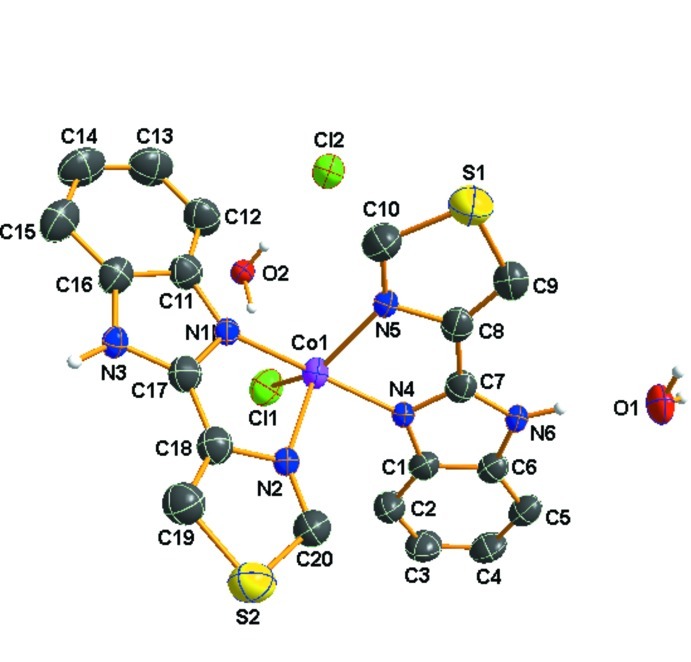
The molecular structure of the title compound, showing 50% probability ellipsoids. H atoms have been omitted for clarity.

**Fig. 2.**
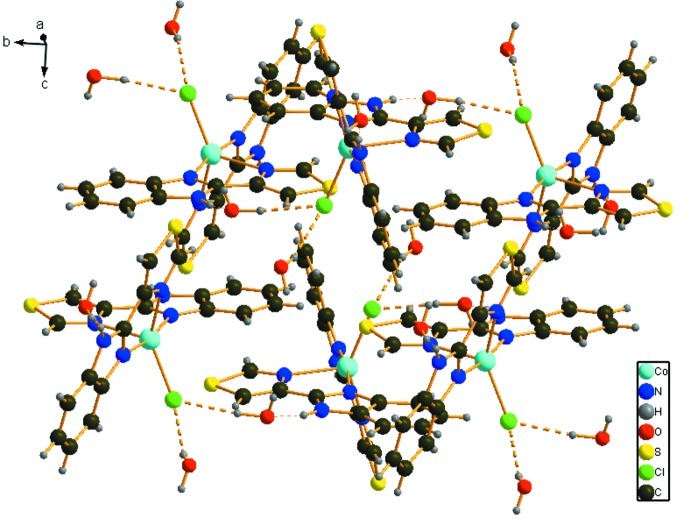
Crystal packing of the title compound, showing the molecules connected by O—H···Cl and N—H···O hydrogen bonds (dashed lines).

## supplementary crystallographic information

### Comment

Metal-based drugs aroused considerable interest in biology and medicine due to their antiproliferative activities. Thiabendazole is an antimicrobial drug belonging to the benzimidazole derivatives and exhibits wide applications in human and veterinary medicine. Thiabendazole had been studied for a long time. The crystal structures of thiabendazole (Trus & Marsh, 1973), thiabendazolium nitrate (Murugesan *et al.*, 1998), thiabendazolium chlorate (Stanley *et al.*, 2002), and a lot of its metal compounds (Devereux *et al.*, 2004, 2007; Flores-Alamo *et al.*, 2010; Jean *et al.*, 2002; Mothilal *et al.*, 2004; Ren *et al.*, 2010; Wisniewski *et al.*, 2001) have been reported.

The title compound consists of a complex cation, a Cl^-^ anion and two uncoordinated water molecules (Fig. 1). The Co^II^ atom is five-coordinated by four N atoms from two chelating 2-(1,3-thiazol-4-yl)-1*H*-benzimidazole ligands and one Cl atom in a distorted trigonal-bipyramidal geometry. In the crystal, N—H···O and O—H···Cl hydrogen bonds (Table 1) and π–π interactions between the thiazole, imidazole and benzene rings [centroid–centroid distances = 3.546 (2), 3.683 (2) and 3.714 (2) Å] link the cations, anions and water molecules into a three-dimensional network.

### Experimental

In a 100 ml flask, thiabendazole hydrochloride (0.237 g, 1 mmol) and CoCl_2_.6H_2_O (0.240 g, 1 mmol) were dissolved in 20 ml water, 20 ml EtOH and 5 ml DMF. The mixture was heated to 350 K for 5 h. After cooling to room temperature, the mixture was filtered and the filtrate was evaporated slowly. After a month, red crystals were collected and washed with water (yield: 0.198 g, 34.9% based on Co).

### Refinement

H atoms bonded to C and N atoms were positioned geometrically and refined using a riding model, with C—H = 0.93 and N—H = 0.86 Å and with *U*_iso_(H) = 1.2*U*_eq_(C, N). Water H atoms were located from a difference Fourier map and refined as riding atoms, with O—H = 0.85 Å and *U*_iso_(H) = 1.2*U*_eq_(O).

### Crystal data

**Table:**

[CoCl(C_10_H_7_N_3_S)_2_]Cl·2H_2_O	*F*(000) = 1156
*M**_r_* = 568.35	*D*_x_ = 1.578 Mg m^−^^3^
Monoclinic, *P*2_1_/*c*	Mo *K*α radiation, λ = 0.71073 Å
Hall symbol: -P 2ybc	Cell parameters from 5625 reflections
*a* = 14.803 (4) Å	θ = 2.5–27.9°
*b* = 11.709 (3) Å	µ = 1.15 mm^−^^1^
*c* = 14.082 (4) Å	*T* = 296 K
β = 101.439 (4)°	Block, red
*V* = 2392.3 (11) Å^3^	0.20 × 0.20 × 0.20 mm
*Z* = 4	

### Data collection

**Table:**

Bruker SMART 1000 CCD diffractometer	4200 independent reflections
Radiation source: fine-focus sealed tube	3517 reflections with *I* > 2σ(*I*)
Graphite monochromator	*R*_int_ = 0.029
φ and ω scans	θ_max_ = 25.0°, θ_min_ = 1.4°
Absorption correction: multi-scan (*SADABS*; Sheldrick, 1996)	*h* = −11→17
*T*_min_ = 0.803, *T*_max_ = 0.803	*k* = −13→13
12683 measured reflections	*l* = −16→16

### Refinement

**Table:**

Refinement on *F*^2^	Primary atom site location: structure-invariant direct methods
Least-squares matrix: full	Secondary atom site location: difference Fourier map
*R*[*F*^2^ > 2σ(*F*^2^)] = 0.033	Hydrogen site location: inferred from neighbouring sites
*wR*(*F*^2^) = 0.096	H-atom parameters constrained
*S* = 1.05	*w* = 1/[σ^2^(*F*_o_^2^) + (0.0452*P*)^2^ + 1.5251*P*] where *P* = (*F*_o_^2^ + 2*F*_c_^2^)/3
4200 reflections	(Δ/σ)_max_ < 0.001
298 parameters	Δρ_max_ = 0.52 e Å^−^^3^
0 restraints	Δρ_min_ = −0.37 e Å^−^^3^

### Special details

**Table:**

Geometry. All e.s.d.'s (except the e.s.d. in the dihedral angle between two l.s. planes) are estimated using the full covariance matrix. The cell e.s.d.'s are taken into account individually in the estimation of e.s.d.'s in distances, angles and torsion angles; correlations between e.s.d.'s in cell parameters are only used when they are defined by crystal symmetry. An approximate (isotropic) treatment of cell e.s.d.'s is used for estimating e.s.d.'s involving l.s. planes.
Refinement. Refinement of *F*^2^ against ALL reflections. The weighted *R*-factor *wR* and goodness of fit *S* are based on *F*^2^, conventional *R*-factors *R* are based on *F*, with *F* set to zero for negative *F*^2^. The threshold expression of *F*^2^ > σ(*F*^2^) is used only for calculating *R*-factors(gt) *etc*. and is not relevant to the choice of reflections for refinement. *R*-factors based on *F*^2^ are statistically about twice as large as those based on *F*, and *R*-factors based on ALL data will be even larger.

### Fractional atomic coordinates and isotropic or equivalent isotropic displacement parameters (Å^2^)

**Table:**

	*x*	*y*	*z*	*U*_iso_*/*U*_eq_	
Co1	0.24191 (2)	0.52351 (3)	0.80442 (2)	0.03535 (12)	
N1	0.12865 (16)	0.48857 (19)	0.70875 (17)	0.0431 (5)	
N2	0.19207 (15)	0.69958 (19)	0.75929 (16)	0.0439 (5)	
N3	−0.00486 (16)	0.54257 (19)	0.62029 (16)	0.0445 (5)	
H3	−0.0486	0.5863	0.5919	0.053*	
N4	0.36009 (15)	0.57629 (19)	0.88293 (17)	0.0433 (5)	
N5	0.32957 (16)	0.4841 (2)	0.70910 (17)	0.0463 (6)	
N6	0.50953 (15)	0.60834 (19)	0.89573 (16)	0.0427 (5)	
H6	0.5629	0.6109	0.8805	0.051*	
O1	0.68432 (15)	0.6368 (2)	0.86990 (18)	0.0747 (7)	
H1A	0.7236	0.6088	0.9164	0.090*	
H1B	0.6967	0.6135	0.8168	0.090*	
O2	0.15760 (14)	0.17298 (19)	0.95087 (16)	0.0608 (6)	
H2A	0.1934	0.1473	0.9156	0.073*	
H2B	0.1578	0.2455	0.9482	0.073*	
S1	0.42099 (6)	0.43913 (9)	0.57955 (7)	0.0668 (3)	
S2	0.14108 (6)	0.90259 (6)	0.71020 (6)	0.0563 (2)	
Cl1	0.18070 (6)	0.45144 (7)	0.93089 (6)	0.0572 (2)	
Cl2	0.29471 (6)	0.07173 (8)	0.83834 (7)	0.0652 (2)	
C1	0.39292 (18)	0.6275 (2)	0.9725 (2)	0.0430 (6)	
C2	0.3493 (2)	0.6578 (3)	1.0480 (2)	0.0565 (8)	
H2	0.2865	0.6460	1.0436	0.068*	
C3	0.4030 (2)	0.7058 (3)	1.1293 (2)	0.0637 (9)	
H3A	0.3757	0.7268	1.1807	0.076*	
C4	0.4968 (2)	0.7237 (3)	1.1363 (2)	0.0573 (8)	
H4	0.5305	0.7565	1.1925	0.069*	
C5	0.5412 (2)	0.6952 (2)	1.0641 (2)	0.0499 (7)	
H5	0.6040	0.7076	1.0695	0.060*	
C6	0.48778 (18)	0.6465 (2)	0.98172 (19)	0.0409 (6)	
C7	0.43232 (18)	0.5669 (2)	0.84080 (19)	0.0407 (6)	
C8	0.41976 (19)	0.5164 (2)	0.7457 (2)	0.0416 (6)	
C9	0.4787 (2)	0.4974 (3)	0.6854 (2)	0.0520 (7)	
H9	0.5414	0.5137	0.6996	0.062*	
C10	0.3214 (2)	0.4410 (3)	0.6220 (2)	0.0599 (8)	
H10	0.2658	0.4138	0.5865	0.072*	
C11	0.08023 (19)	0.3911 (2)	0.6733 (2)	0.0436 (6)	
C12	0.1039 (2)	0.2764 (3)	0.6844 (2)	0.0547 (7)	
H12	0.1600	0.2529	0.7214	0.066*	
C13	0.0396 (3)	0.1989 (3)	0.6377 (3)	0.0637 (9)	
H13	0.0535	0.1214	0.6428	0.076*	
C14	−0.0449 (3)	0.2327 (3)	0.5835 (2)	0.0628 (9)	
H14	−0.0865	0.1772	0.5548	0.075*	
C15	−0.0687 (2)	0.3454 (3)	0.5711 (2)	0.0545 (8)	
H15	−0.1250	0.3680	0.5339	0.065*	
C16	−0.00419 (19)	0.4249 (2)	0.61717 (19)	0.0425 (6)	
C17	0.07502 (18)	0.5765 (2)	0.67555 (19)	0.0403 (6)	
C18	0.10696 (18)	0.6918 (2)	0.69648 (19)	0.0423 (6)	
C19	0.0698 (2)	0.7934 (2)	0.6639 (2)	0.0521 (7)	
H19	0.0132	0.8024	0.6221	0.063*	
C20	0.2169 (2)	0.8065 (2)	0.7725 (2)	0.0484 (7)	
H20	0.2718	0.8284	0.8128	0.058*	

### Atomic displacement parameters (Å^2^)

**Table:**

	*U*^11^	*U*^22^	*U*^33^	*U*^12^	*U*^13^	*U*^23^
Co1	0.02582 (19)	0.0390 (2)	0.0398 (2)	−0.00224 (13)	0.00312 (14)	−0.00274 (14)
N1	0.0372 (12)	0.0425 (13)	0.0489 (13)	−0.0005 (10)	0.0068 (10)	0.0016 (10)
N2	0.0379 (12)	0.0443 (13)	0.0486 (13)	0.0001 (10)	0.0065 (10)	−0.0020 (10)
N3	0.0375 (12)	0.0488 (14)	0.0441 (13)	0.0003 (10)	0.0007 (10)	0.0059 (10)
N4	0.0344 (12)	0.0449 (13)	0.0497 (13)	−0.0011 (10)	0.0064 (10)	−0.0044 (10)
N5	0.0364 (12)	0.0513 (14)	0.0491 (14)	−0.0013 (10)	0.0033 (10)	−0.0072 (11)
N6	0.0341 (12)	0.0482 (13)	0.0450 (13)	−0.0057 (10)	0.0055 (10)	0.0002 (10)
O1	0.0463 (13)	0.1013 (19)	0.0761 (16)	−0.0047 (13)	0.0114 (11)	0.0041 (14)
O2	0.0544 (13)	0.0559 (13)	0.0701 (14)	−0.0024 (10)	0.0072 (11)	−0.0076 (11)
S1	0.0620 (5)	0.0828 (6)	0.0582 (5)	−0.0049 (4)	0.0180 (4)	−0.0234 (4)
S2	0.0639 (5)	0.0412 (4)	0.0634 (5)	0.0021 (3)	0.0121 (4)	0.0026 (3)
Cl1	0.0574 (5)	0.0584 (5)	0.0558 (4)	−0.0100 (4)	0.0112 (4)	0.0053 (3)
Cl2	0.0516 (5)	0.0696 (5)	0.0732 (5)	−0.0039 (4)	0.0096 (4)	−0.0142 (4)
C1	0.0428 (15)	0.0374 (14)	0.0481 (15)	−0.0033 (12)	0.0074 (12)	−0.0040 (12)
C2	0.0489 (17)	0.0591 (19)	0.064 (2)	−0.0050 (14)	0.0161 (15)	−0.0134 (15)
C3	0.072 (2)	0.062 (2)	0.060 (2)	−0.0073 (17)	0.0206 (17)	−0.0183 (16)
C4	0.067 (2)	0.0513 (18)	0.0500 (17)	−0.0102 (15)	0.0022 (15)	−0.0114 (14)
C5	0.0502 (17)	0.0418 (16)	0.0540 (17)	−0.0086 (13)	0.0017 (14)	−0.0009 (13)
C6	0.0422 (15)	0.0365 (14)	0.0422 (15)	−0.0023 (11)	0.0040 (12)	0.0009 (11)
C7	0.0383 (14)	0.0383 (14)	0.0441 (15)	−0.0007 (11)	0.0049 (12)	0.0017 (11)
C8	0.0377 (14)	0.0399 (14)	0.0465 (15)	−0.0012 (11)	0.0068 (12)	0.0021 (12)
C9	0.0439 (16)	0.0540 (17)	0.0602 (19)	−0.0032 (13)	0.0155 (14)	−0.0054 (14)
C10	0.0512 (18)	0.070 (2)	0.0560 (19)	−0.0053 (16)	0.0051 (15)	−0.0192 (16)
C11	0.0408 (15)	0.0463 (16)	0.0448 (15)	−0.0032 (12)	0.0111 (12)	0.0000 (12)
C12	0.0535 (18)	0.0493 (17)	0.0613 (19)	0.0034 (14)	0.0110 (15)	0.0033 (14)
C13	0.077 (2)	0.0449 (18)	0.072 (2)	−0.0066 (16)	0.0224 (19)	−0.0007 (15)
C14	0.077 (2)	0.055 (2)	0.0586 (19)	−0.0255 (17)	0.0189 (18)	−0.0063 (15)
C15	0.0516 (18)	0.067 (2)	0.0447 (16)	−0.0163 (15)	0.0076 (13)	−0.0009 (14)
C16	0.0414 (15)	0.0493 (16)	0.0370 (14)	−0.0069 (12)	0.0080 (12)	0.0004 (12)
C17	0.0375 (14)	0.0443 (15)	0.0394 (14)	−0.0005 (12)	0.0081 (11)	0.0021 (11)
C18	0.0411 (15)	0.0460 (16)	0.0390 (14)	0.0004 (12)	0.0064 (12)	0.0006 (12)
C19	0.0536 (17)	0.0484 (17)	0.0523 (17)	0.0048 (14)	0.0051 (14)	0.0033 (13)
C20	0.0451 (16)	0.0449 (17)	0.0551 (17)	−0.0025 (13)	0.0100 (13)	−0.0057 (13)

### Geometric parameters (Å, º)

**Table:**

Co1—N1	1.974 (2)	C1—C6	1.402 (4)
Co1—N4	1.974 (2)	C2—C3	1.377 (4)
Co1—N5	2.095 (2)	C2—H2	0.9300
Co1—N2	2.239 (2)	C3—C4	1.390 (5)
Co1—Cl1	2.3120 (9)	C3—H3A	0.9300
N1—C17	1.327 (4)	C4—C5	1.357 (4)
N1—C11	1.387 (4)	C4—H4	0.9300
N2—C20	1.308 (4)	C5—C6	1.391 (4)
N2—C18	1.391 (3)	C5—H5	0.9300
N3—C17	1.340 (3)	C7—C8	1.442 (4)
N3—C16	1.379 (4)	C8—C9	1.351 (4)
N3—H3	0.8600	C9—H9	0.9300
N4—C7	1.326 (3)	C10—H10	0.9300
N4—C1	1.393 (3)	C11—C12	1.389 (4)
N5—C10	1.310 (4)	C11—C16	1.397 (4)
N5—C8	1.385 (4)	C12—C13	1.383 (4)
N6—C7	1.338 (3)	C12—H12	0.9300
N6—C6	1.387 (3)	C13—C14	1.388 (5)
N6—H6	0.8600	C13—H13	0.9300
O1—H1A	0.8500	C14—C15	1.368 (5)
O1—H1B	0.8500	C14—H14	0.9300
O2—H2A	0.8500	C15—C16	1.398 (4)
O2—H2B	0.8500	C15—H15	0.9300
S1—C10	1.698 (3)	C17—C18	1.442 (4)
S1—C9	1.707 (3)	C18—C19	1.352 (4)
S2—C19	1.702 (3)	C19—H19	0.9300
S2—C20	1.704 (3)	C20—H20	0.9300
C1—C2	1.395 (4)		
			
N1—Co1—N4	170.18 (10)	C6—C5—H5	121.7
N1—Co1—N5	93.86 (9)	N6—C6—C5	132.0 (3)
N4—Co1—N5	80.51 (9)	N6—C6—C1	105.6 (2)
N1—Co1—N2	79.03 (9)	C5—C6—C1	122.4 (3)
N4—Co1—N2	94.37 (9)	N4—C7—N6	112.5 (2)
N5—Co1—N2	103.42 (9)	N4—C7—C8	118.9 (2)
N1—Co1—Cl1	92.32 (7)	N6—C7—C8	128.6 (2)
N4—Co1—Cl1	97.01 (7)	C9—C8—N5	114.6 (3)
N5—Co1—Cl1	143.16 (7)	C9—C8—C7	132.1 (3)
N2—Co1—Cl1	113.41 (6)	N5—C8—C7	113.2 (2)
C17—N1—C11	106.3 (2)	C8—C9—S1	109.9 (2)
C17—N1—Co1	116.58 (19)	C8—C9—H9	125.0
C11—N1—Co1	136.43 (19)	S1—C9—H9	125.0
C20—N2—C18	110.3 (2)	N5—C10—S1	114.1 (2)
C20—N2—Co1	140.7 (2)	N5—C10—H10	122.9
C18—N2—Co1	109.04 (17)	S1—C10—H10	122.9
C17—N3—C16	107.7 (2)	N1—C11—C12	130.9 (3)
C17—N3—H3	126.2	N1—C11—C16	108.1 (2)
C16—N3—H3	126.2	C12—C11—C16	121.0 (3)
C7—N4—C1	105.8 (2)	C13—C12—C11	116.5 (3)
C7—N4—Co1	115.27 (18)	C13—C12—H12	121.7
C1—N4—Co1	138.82 (18)	C11—C12—H12	121.7
C10—N5—C8	110.9 (2)	C12—C13—C14	122.3 (3)
C10—N5—Co1	136.9 (2)	C12—C13—H13	118.8
C8—N5—Co1	112.05 (18)	C14—C13—H13	118.8
C7—N6—C6	107.6 (2)	C15—C14—C13	121.8 (3)
C7—N6—H6	126.2	C15—C14—H14	119.1
C6—N6—H6	126.2	C13—C14—H14	119.1
H1A—O1—H1B	108.7	C14—C15—C16	116.6 (3)
H2A—O2—H2B	108.7	C14—C15—H15	121.7
C10—S1—C9	90.38 (15)	C16—C15—H15	121.7
C19—S2—C20	89.78 (15)	N3—C16—C11	106.0 (2)
N4—C1—C2	131.9 (3)	N3—C16—C15	132.2 (3)
N4—C1—C6	108.4 (2)	C11—C16—C15	121.8 (3)
C2—C1—C6	119.7 (3)	N1—C17—N3	111.9 (2)
C3—C2—C1	117.4 (3)	N1—C17—C18	120.4 (2)
C3—C2—H2	121.3	N3—C17—C18	127.7 (2)
C1—C2—H2	121.3	C19—C18—N2	114.5 (3)
C2—C3—C4	121.6 (3)	C19—C18—C17	131.3 (3)
C2—C3—H3A	119.2	N2—C18—C17	114.2 (2)
C4—C3—H3A	119.2	C18—C19—S2	110.6 (2)
C5—C4—C3	122.4 (3)	C18—C19—H19	124.7
C5—C4—H4	118.8	S2—C19—H19	124.7
C3—C4—H4	118.8	N2—C20—S2	114.9 (2)
C4—C5—C6	116.5 (3)	N2—C20—H20	122.5
C4—C5—H5	121.7	S2—C20—H20	122.5
			
N5—Co1—N1—C17	−110.9 (2)	C10—N5—C8—C9	0.1 (4)
N2—Co1—N1—C17	−7.97 (19)	Co1—N5—C8—C9	−177.3 (2)
Cl1—Co1—N1—C17	105.44 (19)	C10—N5—C8—C7	178.7 (3)
N5—Co1—N1—C11	80.0 (3)	Co1—N5—C8—C7	1.3 (3)
N2—Co1—N1—C11	−177.0 (3)	N4—C7—C8—C9	178.9 (3)
Cl1—Co1—N1—C11	−63.6 (3)	N6—C7—C8—C9	−0.7 (5)
N1—Co1—N2—C20	−174.8 (3)	N4—C7—C8—N5	0.6 (4)
N4—Co1—N2—C20	−2.2 (3)	N6—C7—C8—N5	−179.0 (3)
N5—Co1—N2—C20	−83.5 (3)	N5—C8—C9—S1	0.6 (3)
Cl1—Co1—N2—C20	97.4 (3)	C7—C8—C9—S1	−177.6 (3)
N1—Co1—N2—C18	4.89 (17)	C10—S1—C9—C8	−0.9 (3)
N4—Co1—N2—C18	177.54 (17)	C8—N5—C10—S1	−0.9 (4)
N5—Co1—N2—C18	96.27 (18)	Co1—N5—C10—S1	175.71 (17)
Cl1—Co1—N2—C18	−82.88 (17)	C9—S1—C10—N5	1.0 (3)
N5—Co1—N4—C7	2.24 (19)	C17—N1—C11—C12	179.2 (3)
N2—Co1—N4—C7	−100.7 (2)	Co1—N1—C11—C12	−11.0 (5)
Cl1—Co1—N4—C7	145.09 (19)	C17—N1—C11—C16	0.4 (3)
N5—Co1—N4—C1	178.8 (3)	Co1—N1—C11—C16	170.2 (2)
N2—Co1—N4—C1	75.9 (3)	N1—C11—C12—C13	−179.0 (3)
Cl1—Co1—N4—C1	−38.4 (3)	C16—C11—C12—C13	−0.3 (4)
N1—Co1—N5—C10	−6.5 (3)	C11—C12—C13—C14	−0.9 (5)
N4—Co1—N5—C10	−178.4 (3)	C12—C13—C14—C15	1.7 (5)
N2—Co1—N5—C10	−86.2 (3)	C13—C14—C15—C16	−1.1 (5)
Cl1—Co1—N5—C10	92.5 (3)	C17—N3—C16—C11	−0.1 (3)
N1—Co1—N5—C8	170.01 (19)	C17—N3—C16—C15	179.9 (3)
N4—Co1—N5—C8	−1.89 (18)	N1—C11—C16—N3	−0.2 (3)
N2—Co1—N5—C8	90.38 (19)	C12—C11—C16—N3	−179.1 (3)
Cl1—Co1—N5—C8	−90.9 (2)	N1—C11—C16—C15	179.8 (2)
C7—N4—C1—C2	−179.5 (3)	C12—C11—C16—C15	0.9 (4)
Co1—N4—C1—C2	3.8 (5)	C14—C15—C16—N3	179.8 (3)
C7—N4—C1—C6	−0.7 (3)	C14—C15—C16—C11	−0.2 (4)
Co1—N4—C1—C6	−177.4 (2)	C11—N1—C17—N3	−0.4 (3)
N4—C1—C2—C3	178.7 (3)	Co1—N1—C17—N3	−172.59 (17)
C6—C1—C2—C3	0.0 (4)	C11—N1—C17—C18	−177.6 (2)
C1—C2—C3—C4	0.1 (5)	Co1—N1—C17—C18	10.2 (3)
C2—C3—C4—C5	0.0 (5)	C16—N3—C17—N1	0.3 (3)
C3—C4—C5—C6	−0.1 (5)	C16—N3—C17—C18	177.3 (3)
C7—N6—C6—C5	179.0 (3)	C20—N2—C18—C19	0.0 (3)
C7—N6—C6—C1	−1.5 (3)	Co1—N2—C18—C19	−179.8 (2)
C4—C5—C6—N6	179.6 (3)	C20—N2—C18—C17	178.4 (2)
C4—C5—C6—C1	0.2 (4)	Co1—N2—C18—C17	−1.4 (3)
N4—C1—C6—N6	1.4 (3)	N1—C17—C18—C19	172.7 (3)
C2—C1—C6—N6	−179.7 (3)	N3—C17—C18—C19	−4.0 (5)
N4—C1—C6—C5	−179.1 (2)	N1—C17—C18—N2	−5.4 (4)
C2—C1—C6—C5	−0.1 (4)	N3—C17—C18—N2	177.9 (2)
C1—N4—C7—N6	−0.3 (3)	N2—C18—C19—S2	0.6 (3)
Co1—N4—C7—N6	177.35 (18)	C17—C18—C19—S2	−177.5 (2)
C1—N4—C7—C8	−179.9 (2)	C20—S2—C19—C18	−0.7 (2)
Co1—N4—C7—C8	−2.3 (3)	C18—N2—C20—S2	−0.6 (3)
C6—N6—C7—N4	1.2 (3)	Co1—N2—C20—S2	179.15 (17)
C6—N6—C7—C8	−179.2 (3)	C19—S2—C20—N2	0.8 (2)

### Hydrogen-bond geometry (Å, º)

**Table:**

*D*—H···*A*	*D*—H	H···*A*	*D*···*A*	*D*—H···*A*
N3—H3···O2^i^	0.86	1.90	2.748 (3)	169
N6—H6···O1	0.86	1.86	2.704 (3)	168
O1—H1*B*···Cl2^ii^	0.85	2.27	3.104 (3)	169
O1—H1*A*···Cl1^iii^	0.85	2.43	3.272 (3)	169
O2—H2*B*···Cl1	0.85	2.45	3.296 (3)	171
O2—H2*A*···Cl2	0.85	2.21	3.052 (2)	174

Symmetry codes: (i) −*x*, *y*+1/2, −*z*+3/2; (ii) −*x*+1, *y*+1/2, −*z*+3/2; (iii) −*x*+1, −*y*+1, −*z*+2.
